# The Epigenetic Origin of Aneuploidy

**DOI:** 10.2174/138920208783884883

**Published:** 2008-03

**Authors:** Luis A Herrera, Diddier Prada, Marco A Andonegui, Alfonso Dueñas-González

**Affiliations:** Unidad de Investigación Biomédica en Cáncer (UIBC)-Instituto Nacional de Cancerología (INCan)-Instituto de Investigaciones Biomédicas (IIBM)-Universidad Nacional Autónoma de México (UNAM), Mexico City, Mexico

**Keywords:** Aneuploidy, cancer, epigenetics, chromosome instability.

## Abstract

Theodore Boveri, eminent German pathologist, observed aneuploidy in cancer cells more than a century ago and suggested that cancer cells derived from a single progenitor cell that acquires the potential for uncontrolled continuous proliferation. Currently, it is well known that aneuploidy is observed in virtually all cancers. Gain and loss of chromosomal material in neoplastic cells is considered to be a process of diversification that leads to survival of the fittest clones. According to Darwin’s theory of evolution, the environment determines the grounds upon which selection takes place and the genetic characteristics necessary for better adaptation. This concept can be applied to the carcinogenesis process, connecting the ability of cancer cells to adapt to different environments and to resist chemotherapy, genomic instability being the driving force of tumor development and progression. What causes this genome instability? Mutations have been recognized for a long time as the major source of genome instability in cancer cells. Nevertheless, an alternative hypothesis suggests that aneuploidy is a primary cause of genome instability rather than solely a simple consequence of the malignant transformation process. Whether genome instability results from mutations or from aneuploidy is not a matter of discussion in this review. It is most likely both phenomena are intimately related; however, we will focus on the mechanisms involved in aneuploidy formation and more specifically on the epigenetic origin of aneuploid cells. Epigenetic inheritance is defined as cellular information—other than the DNA sequence itself—that is heritable during cell division. DNA methylation and histone modifications comprise two of the main epigenetic modifications that are important for many physiological and pathological conditions, including cancer. Aberrant DNA methylation is the most common molecular cancer-cell lesion, even more frequent than gene mutations; tumor suppressor gene silencing by CpG island promoter hypermethylation is perhaps the most frequent epigenetic modification in cancer cells. Epigenetic characteristics of cells may be modified by several factors including environmental exposure, certain nutrient deficiencies, radiation, etc. Some of these alterations have been correlated with the formation of aneuploid cells *in vivo*. A growing body of evidence suggests that aneuploidy is produced and caused by chromosomal instability. We propose and support in this manuscript that not only genetics but also epigenetics, contribute in a major fashion to aneuploid cell formation.

## INTRODUCTION

Chromosome number is transmitted with a high rate of fidelity to daughter cells in each cell division. Deviation from normal chromosome number leading to an unbalanced chromosome complement or to any chromosome number that is not an exact multiple of the haploid number is referred to as aneuploidy. In humans, aneuploidy has different clinical consequences depending on the cell type affected. If germinal cells are affected, aneuploidy is responsible for nearly 20% of spontaneous abortions, most frequently within the first semester, affecting mainly women aged >35 years. Nonetheless, aneuploidy affecting certain combinations of sex chromosomes and small chromosomes such as 13, 18, and 21 may be present in products that survive to gestation, although with birth defects, abnormal development, infertility, mental retardation, and early death in some cases. If aneuploidy occurs in somatic cells, it could result in apoptosis or chromosomal instability, the latter strongly associated with generation, aggressiveness, and resistance of cancer cells [1].

The causes of aneuploidy and chromosomal instability remain largely unknown. Many experiments support the hypothesis of a genetic cause of aneuploidy and chromosomal instability; notwithstanding this, it is important to point out that the human genome is contained in chromosomes, whose structure is more than a simple array of genes, and one that determines chromosomal behavior during mitosis and meiosis, distribution of genes to daughter cells a direct consequence of this behavior. In fact, the human genome contains a large proportion of DNA that forms highly repeated short DNA sequences incapable of coding for proteins; this may play a role in the stabilization and normal functioning of chromosomes that can be implicated in the normal segregation process and in prevention of genomic instability. 

It is now accepted that gene expression and chromosome biology are largely affected by epigenetic marks within chromosomes. These marks occur in the chromatin of eukaryotic genomes, are present in both DNA and the associated histones, and are stable throughout rounds of cell division but do not involve changes in the organism’s underlying DNA sequence. Epigenetic changes play a role in the cellular differentiation process, allowing cells to maintain differential gene expression stably despite containing the same genomic material. Epigenetic information is also essential for the formation of heterochromatin, which is highly compacted, not so accessible to transcription and recombination machinery as are other chromosome regions, and forms structured nucleosome arrays. These characteristics of heterochromatin depend on epigenetic marks that include post-translational modifications of histones and DNA methylation. Although for some time the repetitive DNA contained in heterochromatin was considered as “junk”, recent evidence indicates that heterochromatin can also play important roles during chromosome segregation. Therefore, epigenetics is not only important for genome expression, but also for its correct segregation during mitosis and meiosis.

Here, we review the role of DNA methylation in chromosome stability and the impact of modifications on this epigenetic mark in correct chromosome segregation, with special emphasis on cancer cells. This is not only because the majority of experiments have been carried out either in tumor cells or in cancer cell lines, but also due to the importance of aneuploidy and epigenetics in carcinogenesis. Currently, it is well known that aneuploidy is observed in virtually all cancers. Gain and loss of chromosomal material in neoplastic cells is considered a process of diversification that leads to survival of the fittest clones. Cancer, on the other hand, involves both global and gene-specific hypomethylation and hypermethylation, as well as widespread chromatin modifications [2].

## THE CLASSIC CONCEPT OF THE ORIGIN OF ANEUPLOIDY

Induction of aneuploidy has been associated with defects in chromosome segregation resulting from mitotic spindle alterations, centrosome amplifications, cell-cycle checkpoint defects, non-separation of chromatids, and telomere stability, among others. The classical point of view is that these alterations are induced mainly by mutations in some genes that control chromosome segregation. More than 100 genes are expected to cause chromosomal instability when mutated in eukaryotic cells, including genes involved in telomere metabolism, chromatid cohesion, spindle assembly and dynamics, cell-cycle regulation, DNA repair, and checkpoint controls [3]. However, some important genes are not mutated in aneuploid cells, but are expressed aberrantly [4].

Centrosome proteins such as Aurora kinases may contribute to aneuploidy and usually are overexpressed in cancer cell lines. Aurora A overexpression is associated with centrosome amplification and generation of polyploid cells containing multiple centrosomes. Aurora A overexpression also overrides the spindle assembly checkpoint, resulting in arrested mitosis with incomplete cytokinesis and leading to taxol resistance-associated multinucleation [5]. 

Studies with pre-invasive lesions found a high frequency of centrosome defects in addition to spindle abnormalities, correlated with a high rate of progression to cancer [6]. In the same way, low prevalence of centrosome defects was associated with low-grade lesions. Also, centrosome amplifications have been detected in *in situ-*stage breast carcinoma cells, indicating that these may develop early in the neoplastic process [6]. Centrosome amplification can be associated with diverse genes. For instance, centrosome duplication is controlled by Cdk2/cyclin E complex, which is inhibited by p21. Thus, overexpression of cyclin E or p21 inhibition results in centrosome amplification; moreover, dysfunction of p53, a positive p21 regulator, has the same outcome [7]. Plk1 is necessary for centrosome maturation, is overexpressed in many tumors, and correlates with poor prognosis [8].

Failure in the DNA damage response and double-strand break repair can lead to genetic alteration or chromosomal instability. Similarly, defects in cellular response to double-strand breakage result in genetic mutations, gene amplification, and chromosomal aberrations, and are associated with cancer [9]. For example, ATR activation culminates in cell-cycle arrest, apoptosis, and in DNA repair alteration. ATR has been shown to be a critical factor in the maintenance of chromosomal integrity, and its inhibition leads to chromosomal instability and overexpression of fragile sites. ATM and ATR function as key molecules in the DNA damage response, with a strong influence on the control of cell-cycle checkpoints, DNA repair, telomere maintenance, and apoptosis [10]. Many other specific DNA damage response genes and hereditary mutations in genes such as histone H2AX [11], mre11A, CHEK1, BRCA1, and -2 have been associated with chromosomal instability, and have been shown to cause tumor predisposition by initiating chromosomal instability [12-13].

Some experiments suggest that short telomeres could possess a key role in chromosomal instability, as observed in telomerase-negative immortalized cells, which tend to develop a tetraploid cell population [14]. Polyploidization is an event occurring concurrently with gradual loss of individual chromosome copies. Some preneoplastic lesions, such as Barrett’s esophagus [15] and ulcerative colitis [16], have a high frequency of tetraploid cells. In fact, it has been suggested that some aneuploid cancer cells develop through a tetraploid intermediate [17].

The function of the spindle checkpoint is to ensure that all chromosomes are correctly aligned in metaphase cells and properly attached to the mitotic spindle before chromosome separation can proceed. Chromosomally instable cancer cells in tissue culture do not arrest in metaphase when incubated with microtubule-disrupting agents, most likely due to an abnormal mitotic checkpoint. Spindle checkpoint defects also have been demonstrated in head and neck cancer cell lines [18]. Spindle checkpoint gene mutations were initially reported in colon and pancreatic cancer, as well as in a single breast cancer cell line [19]. However, further analyses revealed that mutations in these genes are rather uncommon; therefore, chromosomal instability in tumors is due to other factors. Subsequent investigations have demonstrated aberrant expression of spindle checkpoint genes (over- and sub-expression) rather than chromosomal instability-related mutations [4, 20-22]. Mutations and control of chromosome segregation genes such as hZW10, -ILCH, and hROD have also been identified as playing a role in the stabilization of this structure, and their dysfunction might contribute to the aneuploidy process [13]. 

## DNA METHYLATION AND INDUCTION OF CHROMOSOMAL INSTABILITY

Although there are several important elements involved in the generation and maintenance of epigenetic marks, we will focus on DNA methylation and its contribution to chromosome stability. Excellent reviews on the elements that make up the DNA methylation machinery have been published recently. Nevertheless, we consider it important to describe briefly some of the most relevant characteristics of this process.

DNA methylation is the covalent attachment of a methyl group from S-adenosyl-methionine (SAM) to carbon 5 of the cytosine ring [23]. It is one of the best studied factors involved in cellular epigenetics and is considered an important gear in the chromatin compactation and maintenance process. In constitutive heterochromatin, DNA methylation is directed by post-translational modifications of histones and completed by proteins, such as methyl-binding proteins, which are characterized by a chromodomain that binds specifically to methylated DNA and recruits enzymatic complexes, e.g., histone deacetylases. This model has been extensively reviewed elsewhere [24] and is currently used to explain the perpetuation of epigenetic modifications that derive in constitutive heterochromatin region formation. In facultative heterochromatin and in maintenance methylation, the epigenetic signal lies in the parental strand and corresponds to a methylated cytosine [25].

The enzymes directly involved in DNA methylation are known as DNA-methyltransferases, or Dnmts. Mammals in general express three families of Dnmts: Dnmt1; -2, and -3 [26-27]. All mammalian Dnmts known to date possess a common catalytic domain that is characterized by 10 conserved amino acid motifs implicated in the catalytic function. Dnmt1 and -3 enzymes contain a large N-terminal regulatory domain responsible for the differential function between them.

Dnmt1 is highly expressed in somatic differentiated cells and is responsible for generation of methylation patterns in many daughter cells after differentiation. In other words, this enzyme is responsible for DNA methylation perpetuation in promoter regions of tissue-specific genes, juxtacentromeric satellites, and imprinting control regions, although its work in maintenance methylation may be complemented by the remaining Dnmts [28]. This enzyme recognizes an asymmetric specific methylated sequence for transferring the methyl group to the newly incorporated cytosine. Hemimethylation is an essential process to maintain DNA methylation patterns during cell proliferation, because any mistake—including alterations in methylation machinery and/or methyl group bioavailability—could generate important scarring in the epigenome. It has been shown, with the use of demethylating de novo agents, that hypomethylating stress may induce changes in the methylation pattern that can be conserved even in absence of the substance. 

Dnmt3a, -b, and -L generate methylation, establish the methylation pattern, and are highly expressed in embryonic stage [29]. DNA methylation is conserved throughout a long time period and many cell cycles [30]. An important component of this cascade of events comprises proteins with a chromodomain, called Methyl-CpG-binding proteins, which interact with either histone deacetylases or histone methyltransferases and other enzymatic complexes to create a compacted chromatin [31].

**Fig. (1):**
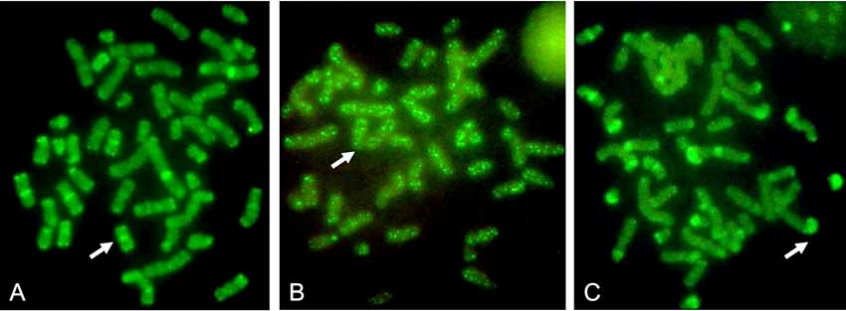
Chromosome methylation patterns of human lymphocytes treated with ethionine or colchicine. Methylation patterns were revealed using a FITC-5methyl-cytosine antibody. A) Normal pattern; B) cells treated with colchicine; C) cells treated with ethionine. Methylation (arrows) is localized in pericentromeric and in some subtelomeric areas in control cultures, while the methylation pattern is localized along the chromosomes in colchicine-treated cells, or in subtelomeric areas in ethionine-treated cells.

It is important to emphasize genome structures in somatic cells that are normally DNA methylated. Some of these structures are involved in the process of chromosome segregation and genome stabilization. In our laboratory, we have found that this pattern of methylation, which involves important chromosomal structures, changes with response to chemical compounds (Fig. **1**), with important consequences in the segregation process (unpublished results).

### DNA Methylation and Chromosome Instability

Cells with reduced DNA methylation levels appear to be more susceptible to undergoing chromosomal loss, gain, or rearrangement, probably because hypomethylation reduces chromosomal stability [32]. Experiments performed with embryonic stem cells show that cells suffered global DNA hypomethylation after Dnmt3b inactivation, generating chromosomal instability characterized by aneuploidy, polyploidy, and some forms of chromosomal aberrations such as chromosomal breaks and fusions. DNA hypomethylation was also associated with premature senescence or spontaneous immortalization [33].

Global hypomethylation in male germ cells may also result in meiotic abnormalities [34-35]. DNA demethylating drugs such as 5-azacytidine, a cytidine analogue [36], and its derived 5-aza-2' deoxycytidine, induce progressive increase in micronuclei formation. These demethylating substances lead to chromosomal aberrations such as deletions, chromosome breaks, isochromosome formation, and translocations [37]. In some cases, these substances induce undercondensation and somatic pairing among the constitutive heterocromatin of chromosomes 1, 9, and 16, 90% of these involving pericentromeric regions [38], figures denominated multibranched chromosomes, and delay in the centromere separation sequence [39], generating preferential exclusion of chromosomes 1, 9, and 16 in micronuclei [40-41], as well as uncoiling and recombination in classical satellite-containing constitutive heterochromatin, but not in alpha satellite [42]. 

5-azacytidine, ethionine, and 9-b-D-arabinofuranosyl-adenine-induced hypomethylation increases sister chromatid exchange (SCE) formation in mammalian cells [43]. SCEs occur only after a second cell cycle following the demethylating pulse, indicating that the key event comprises demethylation of the old parental DNA strand. SCE persisting for many cell cycles after removal of the demethylating pulse may be due to methylation maintenance mechanisms [44]. 

At our laboratory, we have found that in cells exposed to aneugenic compounds, the chromosomal pattern of methylation changes, with an important reduction of the pericentromeric normal methylation that has been correlated with aneuploid cell formation. Changes in the methylation pattern of chromosomes has been previously described in Epstein-Barr-virus-transformed monocytes [45] and in sodium arsenite-treated immortalized cells [46]; nonetheless, its relationship with aneuploidy has not been evaluated. 

### DNA Methylation in Sub-Telomeric and Telomeric Regions

Telomeres are nucleoproteic structures at the end of chromosomes that consist of tandem repeats of the TTAGGG sequence, which is bound by associated proteins [47]. Telomere length is regulated by telomerase, a reverse transcriptase, by alternative telomere lengthening and by histone modifications and sub-telomeric methylation [48]. Telomere function is also controlled by proteins such as TRF1, -2, POT1, TIN2, and Rap1, a mammalian telomeric core complex that forms and protects the telomere [49]. Mammalian telomeres contain histone modifications such as the H3K9 di- and trimethylation produced by histone-methyltransferases Suv39h1 and -2 [50], trimethylation of H4K20 by Suv420h1 and -2, and the retinoblastoma family of proteins, as well as the binding of CBX1, -3, and -5 [51]. 

Sub-telomeric regions are rich in repetitive DNA, have a high density of CpG sequences, and are methylated in human somatic cells [52] observed by cytogenetic methods [53]. The short arms of acrocentric chromosomes 13, 14, 15, 21, and 22 correspond to high concentrations of methylated CCGG repeats at the end of the chromosomes [54]. When epigenetic changes are lost, telomere elongation takes place, suggesting that a compacted chromatin state is fundamental for controlling telomere length. Lack of Dnmts also increases telomeric recombination, indicating that DNA methylation protects the genome from illegitimate recombination between repetitive sequences of telomeres. Loss of methyl groups in telomeres by knock-out of the Dnmt3b has been correlated with the presence of SCE in stem cells [48]. 

Recent reviews analyze the mechanisms implicated in telomere dysfunction-associated chromosome instability formation [49] and the importance of this phenomenon in the stability of the genome; nonetheless, the significance of methylation patterns in sub-telomeric regions in chromosomal instability induction has not been studied yet in depth. Nevertheless, in experiments with ICF cell lines the model of functional depletion of one of the de novo enzymes, Dnmt3b, demonstrate telomeric associations and clonal telomeric rearrangements between chromosomes and anaphase bridges. These are increased by the presence of short dysfunctional telomeres among various chromosomes [55], suggesting that sub-telomeric methylation may be mediated by Dnmt3b. 

Sodium arsenite, a substance associated with DNA demethylation, generates nucleoplasmic bridges and Breakage/Fusion/Bridge (B/F/B) cycles, as well as telomeric associations and dicentric chromosomes, conferring a selective advantage on proliferation associated with an aneuploid state [46]. 

The DNA methylation inhibitor 5-azacytidine has also been able to induce telomeric fusions. Cancer cells commonly possess telomere instability [49, 55-56], and tumor cell lines have lost telomeres at a high rate (10^–6^ events per cell per generation). In tumor cell lines, telomere loss often results in sister chromatid fusion followed by B/F/B cycles [57] lasting for many generations. This inability to terminate B/F/B cycles is likely to contribute to the chromosome instability resulting from telomere loss in human tumor cells. Also, telomere function loss and cell-cycle control disruption are capable of triggering extensive chromosomal instability in colon cancer cells [58]. However, loss of sub-telomeric methylation in cancer and its role in the perpetuation of chromosomal instability in cancer cells has not been studied to date.

### DNA Methylation at Pericentromeric Regions

Pericentromeric regions, the areas surrounding the centromere, are characterized by highly repetitive DNA segments termed classical satellites 2 and 3, which are mainly non-transcribed and highly methylated. These regions contain large amounts of methylated constitutive heterochromatin and are located at metacentric and sub-metacentric chromosomes 1, 9, and 16, and in the short arms of some acrocentric chromosomes such as 13, 14, 15, 21, and 22. Based on ICF syndrome findings, Ehrlich found a relationship between satellite 2 hypomethylation and induction of centromeric decondensation [59]. Pericentromeric region hypomethylation is associated with the induction of a significant reorganization of constitutive pericentromeric heterochromatin [60] and can be clearly observed in cells from patients with ICF syndrome [61].

Somatic cells from patients with ICF exhibit hypomethylated pericentromeric regions associated with chromosomal rearrangements, centromere under-condensation, and the formation of micronuclei preferentially containing chromosomes 1, 9, and 16 [32]. Defects in pericentromeric epigenetic heterochromatin modifications initiate a dynamic HP1-dependent response that rescues pericentromeric heterochromatin function and is essential for viable progression through mitosis [62]. 

Efficient DNA methylation of pericentromeric sequences requires previous trimethylation H3K9 (histone 3 lysine 9) by Suv39h histone methyltransferases [63]. Experimentally induced Suv39h1 overexpression increases trimethylation at H3K9, and is associated with defects in mitotic progression and chromosome segregation [64]. However, mice lacking Suv39h do not exhibit trimethylation at H3K9 within pericentromeric heterochromatin, but rather present increased genomic instability and cancer predisposition [65]. Pericentromeric regions have also been found hypomethylated in some cancers, including hepatocarcinoma, breast, urothelial, and ovarian cancer, and this condition was associated with poor prognosis [66-67]. 

### Chromosome Instability and Aneuploidy Induced by Factors Modifying Epigenetic Marks

One characteristic of epigenetic marks is that they can be modified by genetic and environmental factors, which also induce chromosome instability and aneuploidy. For instance, the ICF syndrome (Immunodeficiency, Centromeric instability, Facial abnormalities), is a rare genetic disease in which some mutations in the Dnmt3b gene have been detected. These mutations not only affect the activity of the enzyme, but also reduce affinity by Dnmt3L [68]. Cells from patients with ICF syndrome present repetitive Alu sequence demethylation [69], satellites 2 and 3 preferentially at pericentromeric regions [70], and hypomethylation of the CpG island of genes located at the inactive X chromosome and of non-satellite DNA repeats D4z4 and NBL2 [71]. These cells also present multiple chromosomal aberrations such as isochromosomes, multibranched chromosomes, and breaks. In addition to the high frequency of chromosomal instability observed in patients with ICF, they do not develop cancer at a higher frequency than the general population, most likely due to their short life span (survival to 10 years of age) [61]. 

Chemical substances such as cadmium, nickel, and sodium arsenite have been categorized as human carcinogens [72], affecting epigenetic marks by different mechanisms. Cadmium chloride induces aneuploidy in MRC-5 human cells [73-74], as well as in other animal models [75-76]. Cadmium inhibits both mammalian and bacterial Dnmt *in vitro* and ex vivo [77-78] in a non-competitive manner, indicating an interaction with the DNA binding domain, probably at the cysteine residue in the active center of Dnmts [79]. This induces DNA hypomethylation early in the cell cycle. Prolonged exposure to cadmium induces signs of cell transformation such as altered cell morphology, increased invasiveness, and increased growth rate accompanied by DNA hypermethylation and enhanced Dnmt activity [78].

Exposure to inorganic arsenic, which interferes with normal methyl- group metabolism, represses expression of DNA methyltransferase genes Dnmt1 and -3a [80]. Sodium arsenite generates hypomethylation and changes in chromosome methylation patterns after acute exposure [46] that are maintained throughout several cell cycles without the substance. Also, arsenic-induced results in c-myc overexpression in TRL 1215 cells is probably associated with many other changes, such as hypomethylation, which is strongly correlated with malignant capacity [81]. Studies in exposed humans found significant DNA hypermethylation of p53 and -16 promoter regions in arsenic-exposed persons [82].

Nickel compounds are potent human and rodent carcinogens [83] and produce chromosome instability and aneuploidy in mammalian cells [84]. The carcinogenic potential of nickel compounds is thought to involve oxidative stress, genomic DNA damage, and epigenetic effects, including gene silencing. Nickel is a DNA methyltransferase activity inhibitor *in vitro* and *in vivo* and induces an initial DNA methylation decrease but with a rebound elevation of total DNA methylation [85]. Water-insoluble nickel compounds induce gene silencing by DNA methylation as a result of de novo methylation, being one of the first models of possible epigenetic carcinogenesis [86]. Short-term exposure of cells to crystalline nickel particles (1–3 days) silences, epigenetically, target genes placed near heterochromatin. A similar effect was found in yeast cells, in which nickel was able to silence the URA-3 gene while it is placed near a telomere silencing element [87-88]. Recently, it was discovered that this change was associated with a decrease in histone H3 and -4 acetylation, as well as an increase in histone H3K9 dimethylation, and that nickel decreased histone demethylase activity [89]. 

Folates are important methyl-group donors for DNA methylation. Nearly two decades ago, it was observed that cells from patients with folate and B_12_ vitamin deficiencies have chromosomal abnormalities that persisted up to 12 months after hematological remission [90]. A study with postmenopausal women in whom moderate folate depletion was induced with a folate-deficient diet demonstrated increased chromosome missegregation frequency in their peripheral blood lymphocytes associated with a decrease on DNA methylation [91]. Similarly, a 3-month 700-µg folate and 7-µg vitamin B_12_ supplementation was associated with a lower frequency of micronuclei in young persons [92]. Recently, it has been suggested that folic acid deficiency may increase not only micronuclei, but also the frequency of nuclear budding and nucleoplasmic bridges, suggesting that folate depletion plays a role in B/F/B cycle initiation [93]. Indeed, some authors have pointed out that folate depletion may be a factor associated with chromosomal instability induction more importantly than mutations in genes such as BRCA1 or -2 [94]. Nevertheless, other group, working with human erythrocytes, reported that folate supplementation did not reduce chromosome missegregation frequency [95]. 

## CONCLUSIONS

The molecular basis of heritable epigenetics and its effects on gene expression have been studied in a variety of organisms and associated with several human diseases. Although it is known that epigenetic marks influence chromatin structure, the potential role of epigenetics in the control of chromosome stability and segregation is still poorly understood. DNA methylation, one of the most important epigenetic marks, influences chromatin dynamics in chromosome areas that are essential for chromosome stability and segregation, such as sub-telomeric and pericentromeric regions, either directly or indirectly through its influence on histone acetylation and histone methylation. The mammalian pattern of DNA methylation acquired during embryogenesis is maintained stably but is potentially affected by the environment; these changes can affect chromosome behavior resulting in alterations in chromosome segregation. The evidence presented strongly suggest an important role of epigenetic marks in the control of chromosome segregation and integrity. Also, considering the importance of aneuploidy in the generation of human pathologies it is important to perform additional investigative efforts to obtain better understanding of the genetic and epigenetic mechanisms that control chromosome integrity and segregation.
